# The General Therapeutic Effect of the Alternative Electric Current of High Frequency and of High Tension

**Published:** 1895-10

**Authors:** G. Apostoli

**Affiliations:** Paris


### THE GENERAL THERAPEUTIC EFFECT OF THE ALTERNATIVE ELECTRIC CURRENT OF HIGH FREQUENCY AND OF HIGH TENSION.

BY DR. G. APOSTOLI, OF PARIS.

Dr. Apostoli, together with Dr. Berlioz, on the 18th of March, 1895, presented a paper on the above-mentioned subject to the Academy of Sciences, of Paris. He now, after longer and riper experience, desires to present a summary statement of his general conclusions:

1° According to Professor d'Arsonval's discoveries, alternative currents of high frequency and of high tension, exert a powerful action upon all living bodies submitted to their inductive influence.

2° The best method of applying these induced currents is to place the patient, free from all contact with electrodes, in the circuit of a large solenoid traversed by these currents.

The patient being thus completely insulated, the currents, which circulate in his body by *auto-conduction*, have their origin in his tissues. The body plays the role of a closed induced circuit.

3° By this method the physiological discoveries of Professor d'Arsonval are confirmed, and we are able to prove the powerful influence of these currents upon the *vaso-motor* system—although they produce absolutely no sensation and although they have no apparent effect upon the motor or sensory nerves.

These currents have, nevertheless, a powerful action upon all the nutritive functions; as has been verified by Dr. d'Arsonval's numerous analyses of the gaseous products of respiration and by Dr. Berlioz's not less numerous analyses of the urinary excreta.

4° The general therapeutic applications to be deduced from this physiological action are confirmed by clinical observation.

Dr. Apostoli has now treated more than a *hundred* patients by this method at his clinic and at his private consultation rooms. The greater number of these patients have been greatly benefited by this new treatment which, be it remarked, has been used to the exclusion of all other forms of medication, dietetic or otherwise.

5° These currents exert in the majority of cases a most powerful and generally beneficial action upon diseases due to *slackening of the nutrition* by accelerating organic exchanges and combustion. This is proved by analyses of the urine made by Dr. Berlioz, of which the following is a brief resume:

The quantity becomes more normal; the products of organic waste are better eliminated.

The *increased combustion* is shown by the diminution of *uric acid*, while the percentage of *urea* is generally increased. The relative proportion of these two substances changes under treatment so as to reach in general the figure of $\frac{1}{40}$.

The elimination of the mineral products is also changed, but in a manner less marked.

6° When daily seances are given, each lasting fifteen minutes, we may generally observe in patients submitted to the influence of these currents the following modifications in their general condition. We mention them in the order of their occurrence:

Return of sleep.

Increase of strength and vital energy.

Increase of gaiety, of power for work and ability to walk.

Improvement of appetite, etc.

In short, *general progressive improvement*.

This general improvement often manifests itself after the first seances before any local influence is apparent and before any change has occurred in the urinary secretions.

7° Local pain and trophic changes are often more slowly affected by these currents and at times they are entirely refractory for a longer or shorter period.

In such cases the same currents must be applied locally by contact with the electrodes.

This subject will be treated later on in a separate communication.

8° The diseases which have appeared incurable by this treatment are those not associated with well-defined organic changes such as *hysteria* and certain forms of *neurasthenia*.

Dr. Apostoli has also observed that certain *localized neuralgias* are refractory to this form of currents, they require its more direct local application.

9° The diseases which have derived most benefit from this therapeutic agent, belong to the *arthritic class: rheumatism* and *gout.*

10° In certain *diabetic* subjects the sugar has disappeared altogether from the urine under the influence of these currents, while in others there has been no such change notwithstanding the manifest and constant improvement in the general condition.

Is this difference due to the imperfection of the electric apparatus or to the manner of its application? It is hoped that further experience will soon afford an answer to this question; although the fact that diabetes has many different causes, may in itself explain the difference in the results obtained by this treatment.

11° In conclusion, the currents of high frequency and of high tension introduced into electro-therapeutics by Dr. d'Arsonval greatly increase the field of action of medical electricity.

They furnish general medicine with a new and valuable means of treatment, capable of modifying more or less profoundly the processes of nutrition.

---

TRIONAL AS A HYPNOTIC.—Spitzer (*Wiener Klinische Wochenschrift*, No. 23, 1895) believes this substance is not only an excellent hypnotic in the different psychoses, but is used with benefit in patients sick with lung and heart disease.

Sleep is produced quickly after the administration of the drug, and continues during the following hours. In some cases, there is a tendency to sleep during the next day, especially in the morning.

In none of his cases could he discover any detrimental action on the heart or lungs. He has noticed, in some cases, a slight disturbance of the digestive tract, but believes it was only an idiosyncrasy.

He believes it approaches morphia in its effects, and may often be substituted for it.—*University Medical Magazine*.

